# The Way Dogs (*Canis familiaris*) Look at Human Emotional Faces Is Modulated by Oxytocin. An Eye-Tracking Study

**DOI:** 10.3389/fnbeh.2017.00210

**Published:** 2017-10-31

**Authors:** Anna Kis, Anna Hernádi, Bernadett Miklósi, Orsolya Kanizsár, József Topál

**Affiliations:** ^1^Institute of Cognitive Neuroscience and Psychology, Research Centre for Natural Sciences, Hungarian Academy of Sciences, Budapest, Hungary; ^2^Department of Ethology, Eötvös University, Budapest, Hungary; ^3^Department of Comparative Biomedicine and Food Science, University of Padova, Padova, Italy

**Keywords:** dog, eye-tracking, oxytocin, emotion, face processing

## Abstract

Dogs have been shown to excel in reading human social cues, including facial cues. In the present study we used eye-tracking technology to further study dogs’ face processing abilities. It was found that dogs discriminated between human facial regions in their spontaneous viewing pattern and looked most to the eye region independently of facial expression. Furthermore dogs played most attention to the first two images presented, afterwards their attention dramatically decreases; a finding that has methodological implications. Increasing evidence indicates that the oxytocin system is involved in dogs’ human-directed social competence, thus as a next step we investigated the effects of oxytocin on processing of human facial emotions. It was found that oxytocin decreases dogs’ looking to the human faces expressing angry emotional expression. More interestingly, however, after oxytocin pre-treatment dogs’ preferential gaze toward the eye region when processing happy human facial expressions disappears. These results provide the first evidence that oxytocin is involved in the regulation of human face processing in dogs. The present study is one of the few empirical investigations that explore eye gaze patterns in naïve and untrained pet dogs using a non-invasive eye-tracking technique and thus offers unique but largely untapped method for studying social cognition in dogs.

## Introduction

In human visual communication the face has a unique function, because it is the most reliable source of one’s emotional or mental states and intentions (Todorov et al., 2008). The ability to recognize behavioral indicators of emotions in others plays a key role in the social organization of group-living species as it might help to predict others’ subsequent behavior. The development of such skills can also be highly beneficial for those sociable domestic animals that live in mixed-species social systems and are commonly kept as companions (Nagasawa et al., 2011; Racca et al., 2012).

Dogs have long coexisted with humans, and have developed a uniquely human-tuned social competence, which, among others, make it possible for dogs to efficiently communicate with humans (for a review see Miklósi and Topál, 2013). Dogs are not only able to detect and recognize the human face (Racca et al., 2010), but also to connect facial expressions with probable outcomes (Nagasawa et al., 2011). Furthermore faces play an important role in how dogs recognize their owners (Adachi et al., 2007; Marinelli et al., 2009). Dogs, similarly to adult humans, show left gaze bias only towards upright positioned human faces but not towards monkey or dog faces or objects (Guo et al., 2009) and they can also learn to discriminate between neutral and happy facial emotional expressions (Deputte and Doll, 2011; Nagasawa et al., 2011). Although this does not necessarily reflect emotion recognition ability in dogs, the finding that they look longer at their owners’ happy vs. sad faces may indicate that dogs are sensitive to human emotional states (Morisaki et al., 2009). Importantly, however, the neuromodulatory mechanisms involved in dogs’ social-emotional receptivity are still largely unexplored.

Several studies have revealed that human socio-cognitive processing is influenced by the neurochemical state of the central nervous system (Kirsch et al., 2005). One of the most prominent neuromodulators is oxytocin, a nine aminoacid long oligopeptide that is produced in the hypothalamus (Lee et al., 2009). Ample evidence suggests that oxytocin influences different aspects of human social behavior (Kosfeld et al., 2005; Buchheim et al., 2009; Heinrichs et al., 2009; Scheele et al., 2012) and it has also been shown to regulate social behavior in many nonhuman species (Lee et al., 2009). According to Guastella et al. (2008) after a single dose of intranasally administered oxytocin people look more to the eye region of human faces. Guastella et al. (2009) also suggest that oxytocin enhances the connection of facial expressions to emotional states. This notion is further confirmed by studies showing that intranasal oxytocin administration selectively increases the recognition ability of certain emotions in humans, although the results are contradictory. While some studies have found an effect regardless of the valence of emotional faces (Domes et al., 2007; Rimmele et al., 2009), in other cases oxytocin only had an effect regarding negative facial emotions such as fear (Fischer-Shofty et al., 2010), anger (Savaskan et al., 2008) and both anger or fear (Kis et al., 2013). The idea that oxytocin differentially modulates human visual attention towards positive or negative facial emotional expressions has been corroborated by an eye-tracking study (Domes et al., 2013) which found that intranasal oxytocin treatment increased gaze to the eye region in case of neutral and happy, but not angry dynamic faces.

The effects of oxytocin on dogs’ social behavior are increasingly explored, and most of the findings support a role of the oxytocin system in dogs’ human-like social skills (for recent reviews, see: Buttner, 2016; Kis et al., 2017). There are some general concerns about peripheral oxytocin measurements (McCullough et al., 2013), and some claims about dog-human co-evolution based on peripheral oxytocin measurements have been widely criticized (Kekecs et al., 2016). This is an ongoing debate, as some authors think that the role of oxytocin in the co-evolution of humans and domestic animals is clear (Herbeck et al., 2016), while others have a more critical attitude towards oxytocin research in dogs (Rault et al., 2017). The literature on the effect of intranasal oxytocin administration to dogs is less controversial, although not only “positive”, e.g., increased ability to follow human pointing, (Oliva et al., 2015; Macchitella et al., 2017), social sensitivity (Kovács et al., 2016b), cognitive bias (Kis et al., 2015), but also “negative”, e.g., less friendly reaction to a threatening owner (Hernádi et al., 2015) effects have been found. This is, however, consistent with human literature suggesting that oxytocin is not a magical “trust elixir” (Mikolajczak et al., 2010), and that despite increasing prosocial behaviors, it does not make people blind to negative social stimuli, but on the contrary in some cases it even increases the salience of negative social stimuli (Theodoridou et al., 2013).

Furthermore recent studies have proved that applying the eye tracking method to dogs is viable (Williams et al., 2011), and it might provide new insights into dogs’ face processing and social-communication skills. It has been found Somppi et al. (2012) that dogs, without any task-specific pre-training, focus their attention on the informative regions of facial images, which support the notion that eye tracking technology offers promising possibilities for studying the effects of oxytocin on visual processing of human emotional expressions in the dog. Using the eye-tracking method it was also proven that dogs follow human gaze if it is preceded by communicative signals directed to them (Téglás et al., 2012). These three research groups that have so far conducted eye-tracking studies on dogs have used different methodological solutions (e.g., head-mounted vs. contact-free eye-tracking, family dogs vs. laboratory dogs, trained vs. untrained dogs), which all come with different advantages. Téglás et al. (2012) was able to collect data from a representative sample of untrained family dogs, Somppi et al. (2012) could achieve sustained attention and long fixation times with purpose-trained laboratory dogs, Williams et al. (2011) developed a method that promises application to real-life situations (as opposed to computer-screen images).

In the present study we capitalized on the eye tracking technology, and set out to address the question whether dogs’ face processing, as measured by subjects’ looking pattern, changes due to the oxytocin treatment and if these changes are specific to certain facial emotion expressions. In order to do so, first we assessed the most adequate presentation method in terms of number of stimuli, to allow dogs to maintain a focused attention. We assessed (Study I) the maximum number of stimuli that could be presented without risking that an order effect would overwrite any other effects of interest. Human faces were presented from both genders and with different emotional expressions in order to determine if these factors have a major effect on dogs’ viewing patterns. Then we used eye-tracking to investigate the effects of a single dose of intranasal oxytocin on pet dogs’ viewing patterns of emotional faces (Study II). We hypothesized that: (1) most looking times will be focused on informative regions (e.g., eyes and mouth) as in previous studies (Somppi et al., 2012); that (2) after oxytocin treatment angry faces will be more salient for dogs (Theodoridou et al., 2013) making them avert gaze from these images; and that (3) oxytocin will increase looking time to the eye region (as in humans, Guastella et al., 2008). Dogs’ age, sex, training level and head shape were also considered as confounding variables.

## Study I

### Background

Previous studies investigating visual processing in dogs presented a very limited number of stimuli both in eye-tracking test (Somppi et al., 2012; Téglás et al., 2012) and in preferential looking (image projection) paradigms (Faragó et al., 2010; Racca et al., 2012; Péter et al., 2013), which raises concerns of pseudoreplication (Lazic, 2010), e.g., the effect found might be specific to those images only and might not generalize to other stimuli. In our first study we aimed to investigate dogs’ visual attention span in a sequential image presentation task in order to determine the maximum number of stimuli that could be presented without a serious order effect (that would potentially mask other effects of interest). In order to do this we presented a sequence of six images of male and female faces expressing happy, angry and fearful emotions.

### Methods

#### Subjects

Fifty-eight adult pet dogs (females/males: 30/28; mean age ± SD: 4.26 ± 3.07; from 25 different breeds and 16 mongrels) were recruited from the Family Dog Project Database built and maintained by Department of Ethology, Eötvös University. In order to be selected for this study the subject had to be naïve to the task, and older than a year. 27 dogs had to be excluded due to subjects’ inattentiveness and/or their head shape (too long nose, lateral position of the eyes) that made the eye-tracker calibration impossible. The final sample consisted of 31 dogs (male/female: 15/16; mean age ± SD = 4.18 ± 2.76; from 15 different breeds and 8 mongrels).

#### Experimental Procedure

The experiments took place in a laboratory room (4 m × 4 m). The eye gaze data was collected with a Tobii X50 Eye Tracker (Stockholm, Sweden) at 50 Hz, that was the same temporal resolution used by a previous dog eye tracker study (Téglás et al., 2012). The eye tracker had 0.5–0.7 degree accuracy 30 × 16 × 20 cm freedom of head movement. The stimuli were presented on a 17-inch LCD monitor positioned behind the eye tracker.

**Figure 1:**
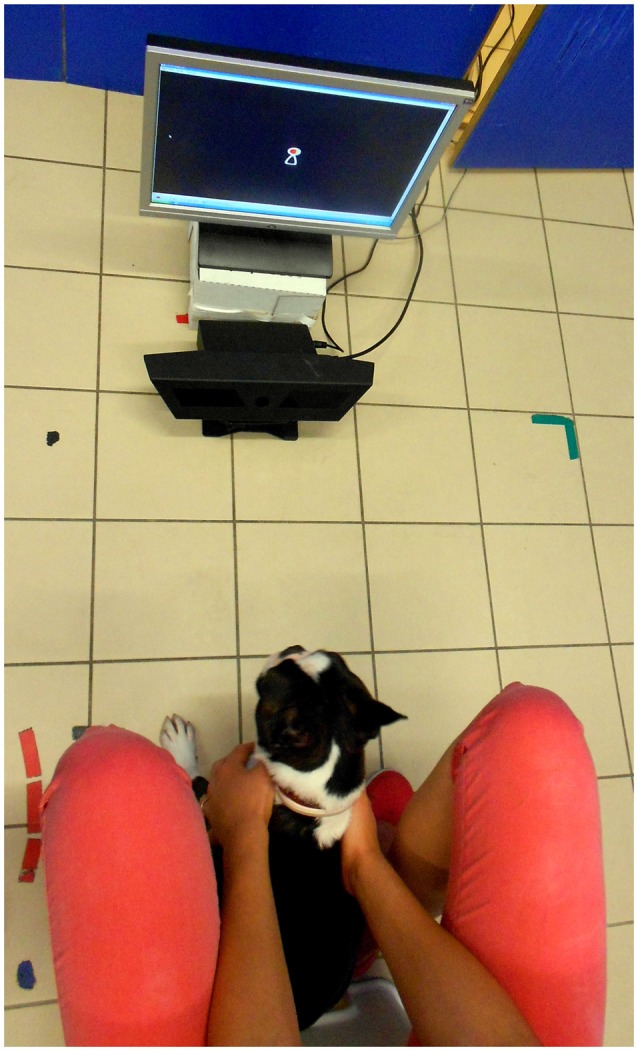
The dog’s position during stimuli presentation.

When the owner and the dog arrived at the laboratory dogs were allowed to freely explore the room and to interact with the experimenter for approximately 5 min. During this time owners were informed in detail about the experimental procedure. Then we checked whether the dog’s eyes could be captured by the Track Status viewer to determine if a subject had the potential to successfully pass the calibration. The experimenter placed a treat on top of the eye tracker and encouraged the dog to take the treat from there. Once the dog became familiar with the equipment the owner was asked to sit the dog in front of the eye tracker and hold the dog by placing both hands on its chest (Figure 1). Depending on the size of the dog the distance of the equipment from the dog varied (approx. 50–80 cm) and the angle was adjusted until the eye-tracker could register both of the dogs’ eyes. During the calibration and stimulus presentation phase the owner did not interfere with the dog nor did he/she force it to watch the screen.

#### Calibration

The eye gaze recording was preceded by a five-point calibration phase. This was run using the ClearView 2.5.1 software package and the procedure was identical to that reported by Téglás et al. (2012). The calibration was considered successful if both of the dog’s eyes were registered on at least four of the five points.

#### Stimulus Presentation

After successful calibration the experimenter left the room and the test trial followed during which Clearview 2.5.1 software presented six images of three different male and three different female faces from the Radboud Faces Database (Langner et al., 2010) showing happy, angry or fearful emotional expressions. The stimulus presentation started with an introductory phase during which an attention getter stimulus (a rattling and moving toy) was presented in the middle of the screen for 4 s. It was followed by the presentation of a face stimulus for 5 s in the middle of the screen. The attention-getter reappeared on the screen between each facial stimuli to redirect the dogs’ attention. The presentation order of the first two facial stimuli was counterbalanced between subjects (first stimulus: angry female, second: happy male, *N* = 12 dogs; first stimulus: happy male, second: angry female, *N* = 19 dogs), while the order of the other stimuli was fixed (third: fearful female, fourth: angry male, fifth: happy female, sixth: fearful male). During the presentation of emotional facial expressions a neutral beep sound was played.

#### Data Analysis

*Gaze duration* was calculated as the time subjects spent looking at the screen during the presentation of the face stimuli. Gaze duration data of the first presented stimuli (mean looking time at the first presented stimuli) was used to test the effects of age (Pearson correlation), training experience (trained vs. untrained dogs; independent samples *t*-test), head shape (short vs. long nose dogs; independent sample *t*-test) as well as the potential differences between male and female subjects (independent samples *t*-test).

Linear Mixed Model (LMM) was used to determine how the presentation order (from first to sixth; within subjects covariate), as well as the emotional expression (happy, angry, fearful; within subject factor) and the gender (male or female; within subject factor) of the stimuli faces influenced the total gaze duration towards the screen.

Based on the results of the first model (see later) data of the first two faces (angry female and happy male, in a counterbalanced order across subjects) was entered in another model (LMM) in order to test the effects of order (first/second, within-subjects factor), angry female/happy male (within-subjects factor) and their interaction. As a strong order effect was found across the six images, with this second model we aimed to see if restricting the analysis to two images only would yield different results.

Data of the first image was used to test how long dogs look into the different regions of the face. Each stimulus face was divided into four *AOIs*: eyes, mouth, forehead and neck regions. The size of AOIs for the eye, mouth and forehead were the same for all faces, the neck AOI was 33% smaller. Gaze durations were calculated for each of the AOIs. Then *gaze preference scores* were calculated for each dog based on the gaze duration data: we ranked the four facial AOI according to their efficiency in attracting a subject’s attention by assigning rank 1 to the lowest value, and assigning the mean of ranks to ties. In order to correct for the fact that the neck region was 33% smaller, data from this region was multiplied by 1.5 before the rank transformation. Friedman test was used to test if dogs preferred to look at one region over another (we first tested for the data pooled together for all subjects, and then tested separately the looking pattern for the two different images).

### Results

Gaze duration toward the first stimuli was not affected by the dog’s sex (*t*_(29)_ = 0.65; *p* = 0.52), age (Pearson *r*_(29)_ = −0.22; *p* = 0.26), training experience (*t*_(15,2)_ = 1.48; *p* = 0.16) and head shape (*t*_(5,29)_ = 1.23; *p* = 0.27).

**Figure 2:**
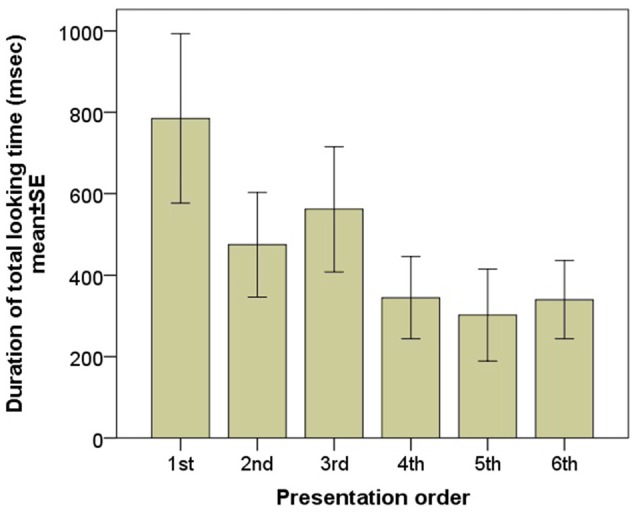
Gaze duration of the subjects during the six consecutive image presentations.

According to the LMM there was a significant main effect of the sequence of presentation on mean gaze duration towards the screen indicating a strong decrease in viewing duration (*F*_(1)_ = 8.743, *p* = 0.004; Figure 2). No effect of emotional expression (happy vs. angry vs. fearful; *F*_(2)_ = 1.287, *p* = 0.287) and gender (*F*_(1)_ = 0.869, *p* = 0.3521) was found.

When only data of the first two images entered in the LMM the order effect also disappears (first/second: *F*_(1)_ = 1.329, *p* = 0.254; angry female/happy male *F*_(1)_ = 0.286, *p* = 0.595; order × image interaction: *F*_(1)_ = 0.449, *p* = 0.505).

**Figure 3:**
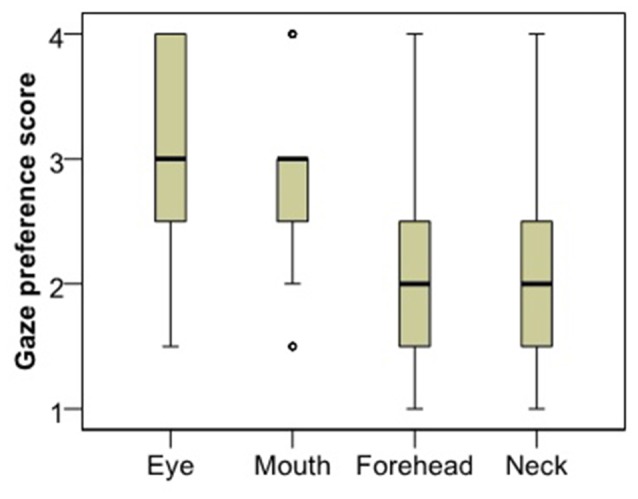
Viewing preference of the different face regions as expressed in the rank of viewing times.

Analysis of gaze preference scores for the first image (Figure 3) showed that dogs differentiate between the facial regions in their looking pattern (*χ*^2^ = 24.260, *p* < 0.001). They look more to the eye region compared to both the neck (Dunn *post hoc*, *p* < 0.001) and the forehead region (*p* = 0.003); and they look more to the mouth compared to the neck (*p* < 0.001), although not the forehead (*p* = 0.084). There was no difference between the eye and the mouth (*p* = 0.240) or the neck and the forehead (*p* = 0.098) regions. The same result remained both for subjects that viewed the angry female image (*χ*^2^ = 12.108, *p* = 0.007) and those who viewed the happy male image (*χ*^2^ = 12.770, *p* = 0.005).

### Discussion

This pilot study investigated how certain properties of human faces e.g., gender, emotional expression and sequence of the presented faces influence the dogs’ looking behavior. We found that the presentation order had an important effect on the dogs gaze duration toward the screen, that confirms the study design of previous studies using both eye-tracking (Somppi et al., 2012; Téglás et al., 2012) and projected images (Faragó et al., 2010; Racca et al., 2012) and suggests that due to the limited attention span of dogs fewer stimuli should be used. We found no influence of the model’s gender and there was no difference in the gaze duration toward faces expressing different emotional expressions either. This is somewhat in contrast with previous studies suggesting that dogs recognize the gender of humans (Wells and Hepper, 1999; Deputte and Doll, 2011) as well as the different emotions (Morisaki et al., 2009; Deputte and Doll, 2011; Nagasawa et al., 2011). This difference might be due to special circumstances that dogs face whilst participating in an eye tracking experiment (e.g., watching a computer screen without a task might not be a natural behavior for a dog). Note also that while previous studies coded the dogs’ behavior/head movement (Morisaki et al., 2009; Deputte and Doll, 2011) or used touch screen technique (Nagasawa et al., 2011), here we measured gaze durations, a more specific indicator of attentional engagement. It is also possible that the strong order effect that we found masked other more subtle effects, although the fact that we found no effect in the model that analyzed the first two images (angry female vs. happy male) makes this explanation somewhat less likely. Our results are also in line with the notion (Somppi et al., 2012) that dogs show a greater visual preference for emotionally meaningful face areas (e.g., the eyes as opposed to the neck and the forehead).

## Study II

### Background

Based on the results obtained in Study 1, we designed the second study that aimed to test the effect of intranasal oxytocin treatment on dogs’ human face and emotion processing. As no effect of image gender was found, we decided to restrict our stimuli to one gender only. In order to minimize the confound arising from order effects only two stimulus images were used for longer presentation duration (7000 ms). Although no effect of emotion was found in Study I, we decided to use both happy and angry facial expressions as stimuli, due to the extended human literature showing an emotion-specific effect of oxytocin on face processing (Domes et al., 2007; Savaskan et al., 2008; Guastella et al., 2009; Marshall-Pescini et al., 2009; Fischer-Shofty et al., 2010; Kis et al., 2013).

### Methods

#### Subjects

A total of 125 family dogs naïve to the experimental setting were recruited on a voluntary basis from the Family Dog Project (Abdai and Miklósi, 2015) database. Of these 48 dogs were excluded as their eyes could not be captured by the eye tracking device due to subjects’ inattentiveness and/or their head shape (too long nose, lateral position of the eyes). The remaining 77 dogs received either placebo (PL group, *N* = 32 dogs) or oxytocin (OT group, *N* = 45 dogs) pretreatment. However, further 31 dogs (8 in the PL and 23 in the OT groups) had to be excluded because they did not provide eye gaze data for both of the stimuli pictures. Surprisingly, oxytocin pre-treated dogs had to be excluded in a much higher ratio than was the case for both previous studies and placebo treated dogs in the present study. One possible explanation is that as oxytocin has an effect on pupil dilatation (especially when viewing emotional stimuli; e.g., Leknes et al., 2013), this might underlie the high drop-out rate we experienced (e.g., changes in dog’s pupil size caused that the eye-tracker did not record valid gaze data in some cases).

The final sample consisted of 46 subjects from 20 different breeds and 10 mongrels; *N* = 24 in the Placebo (mean age ± SD: 4.52 ± 2.23; females/males: 10/14) and *N* = 22 in the Oxytocin (mean age ± SD: 4.31 ± 2.5; females/males: 8/14) groups.

#### Pre-Treatment

If the eye tracker was able to detect both eyes of the dog a single intranasal dose of oxytocin (Syntocinon-Spray, Novartis) or placebo (isotonic natriumchlorid 0.9% solution) was administered. The amount of solution sprayed into nostrils depended on the dogs’ body size: large and medium sized dogs (over 18 kg) received 12 IU (1 and 2 puffs per nostril), small dogs (under 18 kg) received 8 IU (1-1 puff per nostril). Treatment was followed by a waiting period of 40 min (similarly to human experiments; e.g., MacDonald et al., 2011) presumed to be necessary for intranasally administered neuropeptides to develop their effect on the central nervous system (Born et al., 2002). This pre-treatment procedure has been validated for dogs by showing that oxytocin as compared to placebo decreases heart rate and increases heart rate variability (Kis et al., 2014) and was used in several studies that yielded behavioral differences between oxytocin vs. placebo pre-treated dogs (Hernádi et al., 2015; Kis et al., 2015; Kovács et al., 2016a,b).

#### Calibration

After the waiting period the dog-owner dyad entered the laboratory again and the owner was asked to set her dog into the testing position. The eye gaze recording was preceded by the same five-point calibration process used in Study I (section “Study I: Methods: Calibration”).

#### Stimulus Presentation

After the successful calibration the experimenter left the room and the test trial followed during which Clearview 2.5.1 software presented two images of male faces expressing two different emotions (happy and angry). Stimulus presentation started with an introductory phase during which an attention getter (a rattling and moving toy) was present on the screen for 4 s—in order to direct the dogs’ attention to the center of the screen. It was followed by the presentation of a happy or angry face for 7 s displayed on either the left or the right side of the screen (We presented images to the left and right side in order to avoid that the fixating to the attention getter, presented to the middle immediately preceding the stimuli, causes fixations to relevant target regions). Then the whole presentation procedure was repeated (attention-getter stimulus for 4 s and facial image for 7 s) in this case the location (left or right) and the emotional expression (angry or happy) were reversed.

The stimulus material included facial photographs of four male individuals from the Radboud Faces Database (Langner et al., 2010). Images were randomly selected from the 20 Caucasian adult males the database contained. Models wore black t-shirts, had no hair on the face and wore no glasses, makeup or jewellery. The chosen images all showed the emotional expression with eyes directed straight ahead and from a 90° camera angle. Photos had been corrected for white-balance, and spatially aligned according to facial landmarks. We did not make any modification to the images obtained from the database. All images were of an original size of 1024 × 681 pixels, and in our monitor were presented in the size of 26 cm × 17.3 cm. The images were randomly assigned to the dogs during the test with the restriction that each dog would see the different emotional expressions of the same person. The type (happy or angry) and the location (left or right side) of the firstly presented images were counterbalanced between subjects in both OT and PL groups. During the presentation of emotional facial expression neutral beep sound was played.

#### Data Analysis

Due to a generally low duration that dogs spent looking at the stimulus (see “Results” section), only gaze duration data could be analyzed, but not the number of fixations. The 200 ms criteria commonly used for human infant eye-tracking (Gredebäck et al., 2009) would result in zero fixations for a considerable proportion of dogs. While some studies address this problem by lowering the fixation threshold for dogs to 0 or 75 ms we decided to use only the gaze durations instead, as it is hard to argue that a fixation of 0 ms is meaningful.

*Gaze duration* was calculated as the time subjects spent looking at the screen during the presentation of the stimuli. Each stimulus face was divided into four *AOIs*: eyes, mouth, forehead and neck regions. The size of AOIs for the eye, mouth and forehead were the same for all faces, the neck AOI was 33% smaller. We summed up these AOIs to get a whole face region as well. Gaze durations were calculated for each of the AOIs. The *relative gaze durations* toward eye, mouth, forehead, neck and whole face regions were calculated by dividing the means of the gazing time toward these regions by the means of the total gazing time at the screen. Then *gaze preference scores* were calculated for each dog based on the gaze duration data: we ranked the four facial AOI according to their efficiency in attracting a subject’s attention by assigning rank 1 to the lowest value, and assigning the mean of ranks to ties. In order to correct for the fact that the neck region was 33% smaller, data from this region was multiplied by 1.5 before the rank transformation.

Gaze duration data (mean looking time at the two presented stimuli) was used to test the effects of age (Pearson correlation), training experience (trained vs. untrained dogs; independent samples *t*-test), head shape (short vs. long nose; independent samples *t*-test) as well as the potential differences between male and female subjects (independent samples *t*-test). LMM was used to determine how the treatment (OT or PL; between subjects factor), as well as the emotional expression (happy or angry; within subject factor) and the presentation order (first or second; within subject factor) of the stimuli influenced the relative gaze durations towards the different AOIs.

*Gaze preference scores* were used to test if dogs in the OT and PL groups have any preference for a designated facial region of the happy/angry faces (Friedman test, Dunn *post hoc* test). For the statistical analysis the SPSS 18.0 statistical package and InStat software were used.

### Results

There was no difference in age (*t*_(117)_ = 0.39; *p* = 0.69), gender ($\chi_{\left(1\right)}^{2}$ = 1.23; *p* = 0.27) and training experiences ($\chi_{\left(1\right)}^{2}$ = 0.27; *p* = 0.604) when comparing dogs who successfully passed to those who failed to pass the calibration. Dogs in the final sample looked at the screen on average 19.7% (2759.54 ms) of the total (2 × 7000 ms) time (ranged between: 80–10,508 ms) when the facial images were presented. Gaze duration toward the screen was not affected by the dog’s gender (*t*_(44)_ = 0.15; *p* = 0.88), age (Pearson *r* = 0.009; *p* = 0.95), head shape (*t*_(44)_ = 1.33; *p* = 0.19) and training experience (*t*_(44)_ = 0.29; *p* = 0.77).

There was a significant interaction (LMM; for full models see Supplementary Materials) between emotional expression and sequence of presentation in case of relative gaze to the eye (*F*_(1,84)_ = 7.37; *p* = 0.008) and mouth (*F*_(1,84)_ = 7.54; *p* = 0.007) region. In case of the first stimulus, dogs looked more to the happy face’s eyes than to the angry face’s eyes and more to the angry face’s mouth than to the happy face’s mouth. In contrast in case of the second stimulus, dogs looked more to the angry face’s eyes than to the happy face’s eyes and more to the happy face’s mouth than to the angry face’s mouth. Relative gaze duration to the forehead region was also affected by the sequence of presentation (*F*_(1,84)_ = 3.94; *p* = 0.05). Subjects looked more to the forehead region at the first presented faces than at second one.

**Figure 4:**
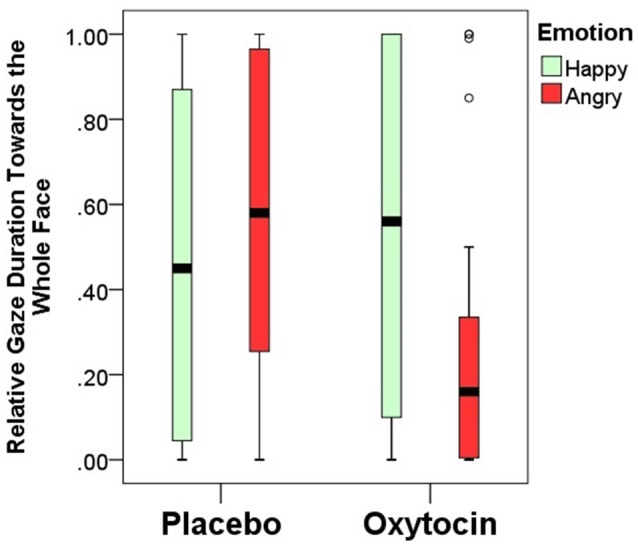
Relative gaze duration (mean ± SE) towards the whole face expressing happy/angry emotion in the placebo and oxytocin groups. Median, quartiles, whiskers, outliers.

Relative gaze duration toward the whole face indicates a significant interaction between emotional expression and pretreatment type (*F*_(1,84)_ = 4.67; *p* = 0.03). After having received intranasal administration of oxytocin, dogs gazed less toward the human face expressing negative, but not positive emotion (Figure 4). Relative gaze durations towards the other face regions (eye, mouth, neck and forehead) were not influenced by the pretreatment or emotional expression and no interaction between pretreatment and emotional expression was found either (all *p* > 0.05; for full models see Supplementary Materials).

**Figure 5:**
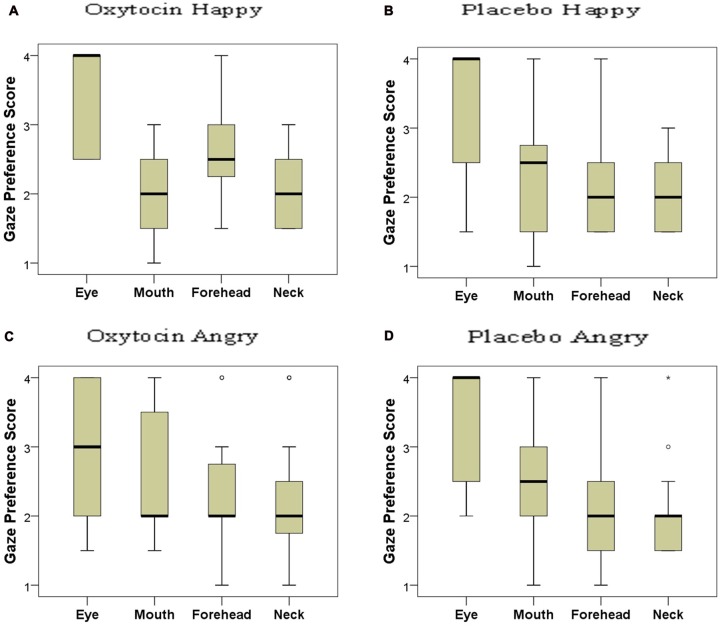
Looking preference of subjects as reflected in their rank scores for happy (**A**: oxytocin, **B**: placebo) and angry (**C**: oxytocin, **D**: placebo) faces. A higher score indicates a higher preference. Median, quartiles, whiskers, outliers.

Based on the distribution of gaze durations toward the different parts of angry and happy faces, the facial regions were ranked and the gaze preference scores for the different AOIs of happy and angry faces in both OT and PL groups were analyzed (Figure 5). In the placebo-treated group, we found significant differences in terms of dogs’ looking patterns for both the happy ($\chi_{\left(3\right)}^{2}$ = 19.705; *p* < 0.001) and the angry ($\chi_{\left(3\right)}^{2}$ = 19.123; *p* < 0.001) facial images. Replicating our results in study 1 dogs preferred to look to the eye region compared to the forehead (Dunn *post hoc* test; *p* < 0.05) and neck region (Dunn *post hoc* test; *p* < 0.01) of both happy and angry faces. A similar attentional bias was found in the oxytocin-treated group for the angry faces ($\chi_{\left(3\right)}^{2}$ = 9.333; *p* = 0.025), although the *post hoc* test did not reach significance. This differential looking pattern, however, was not found in case of the happy ($\chi_{\left(3\right)}^{2}$ = 6.706; *p* = 0.082) faces for the oxytocin-treated group. Directly compared, the oxytocin and the placebo groups did not differ in their rank scores for any of the facial regions (all *p* > 0.05 for both happy and angry faces), see supplementary material.

## General Discussion

In the present study, we investigated visual processing of human faces in dogs and demonstrated differential effects of oxytocin on the eye gaze patterns towards faces expressing positive and negative emotions. Dogs in the control groups (i.e., all subjects receiving no pre-treatment in study 1 and those subjects in the main study that received placebo treatment) displayed a general preference towards the eye region of the human face regardless of valence of the emotional expression.

Our results are also important from the methodological point of view, as they add to the handful of experiments that have so far employed eye-tracking in order to measure gaze patterns in non-human animals (e.g., chimpanzee: Kano and Tomonaga, 2009; Hattori et al., 2010; dog: Williams et al., 2011; Somppi et al., 2012). We confirmed previous claims that eye-tracking can be applied to study task- naïve pet dogs (Téglás et al., 2012). However the large number of subjects that had to be excluded raise some concerns about the representativeness of the subjects participating in these studies and also pose considerable practical difficulties for future research. Despite some general “rules of thumb” (e.g., dogs should have no hair in the eyes) we did not find any factor that would predict successful eye-tracker calibration as no effect of head shape or training experience was found. A possible solution to this methodological problem is to train the dogs to lie still for the purpose of an eye-tracking study. For example, in a recent study in which dogs were specifically trained to meet the requirements of eye-tracking (Somppi et al., 2017) 43 of the 46 recruited subjects successfully completed the experiment. However training dogs for such a task might heavily influence their looking pattern as well as their cognitive processes during image viewing, as training has been shown to modulate attention in general (Vas et al., 2007). Specific trainings (Marshall-Pescini et al., 2009) as well as general training level (Marshall-Pescini et al., 2008) have also been found to influence performance and certain aspects of behavior in social and cognitive tasks The future combination of two approaches would be ideal. It is also important to mention that our study suggests that eye-tracking can only be used with short stimuli presentation as the looking time of dogs quickly decreases over time.

Our findings fit well with the widely held notion that the eye region of another is a strong attention getter for group members in many social species (Emery, 2000). However after oxytocin pre-treatment this preference only remained for the angry but not the happy faces, contrary to human findings where oxytocin increased gaze to the eye region (Guastella et al., 2009). This difference between dogs and humans might be attributed to a difference in the meaning of gaze cues. In humans, staring eyes (establishing eye contact) have two distinct functions as they can signal either competitive (threatening—Wieser et al., 2009) or collaborative (information sharing—Senju and Csibra, 2008) attitudes toward the partner. Although direct gaze in face–to–face situations is commonly used to indicate a positive, information sharing attitude in humans from very early on Csibra (2010), the predominant role of this signal between non-human subjects is evoking fear or aggression and has little (if any) collaborative property. Even among dogs direct gaze is mainly used for signaling dominance and as a form of ritualized aggression (Schenkel, 1967).

Further studies could follow up on the finding that dogs use certain relevant regions of the face to assess emotions by presenting only those parts (e.g., the eyes) of faces with different emotions. A recent touch-screen study (Müller et al., 2015) showed that dogs can generalize from upper half to lower half of the face, but more fine-scaled analysis with eye-tracking technology will add further information. A further interesting question is whether looking patterns for positive and negative faces both differ from those for neutral faces, or if the difference between negative and positive faces can be attributed to only one of them.

Although dogs often use direct gaze for the same purpose as infants do (demanding attention or initializing communicative interaction—Miklósi et al., 2003; Passalacqua et al., 2011) while interacting with their human caregivers or familiar partners, eye contact with an unfamiliar human has the potential to evoke fear (Vas et al., 2005) and to increase symptoms of anxiety (heart rate—Gácsi et al., 2013). In line with this, we may assume that in the test trials the sudden appearance of an unfamiliar human’s face and his staring eyes in a very intimate, face-to-face position was conceived as threatening by the dog, and as a consequence, they showed increased attention towards the eye region of the faces regardless of the displayed emotional expression. It is also worth mentioning that the gaze of negative emotional face is a particularly effective cue to attention also in humans (Holmes et al., 2006) and this is especially true for anxious people who seem to show an attentional bias to threatening faces in an eye tracking experiment (Armstrong et al., 2010).

In the case of dogs treated with oxytocin, however, the analysis of eye gaze patterns provided a somewhat different picture: (1) subjects in OT group generally showed a weaker tendency to look at negative facial images compared to PL group, and, at the same time; (2) the preferential looking to the eye region of happy human faces disappeared. In contrast to this in a study conducted on trained dogs analyzing the number of fixations (Somppi et al., 2017) it was found that dogs after oxytocin treatment fixated less often at the eye region of angry faces and revisited more often the eye region of happy faces. These differences might probably be attributed to the subjects in the two studies being naïve vs. trained for the eye-tracking task. Both findings support the notion that dogs’ gaze bias towards the eye region of faces can be regarded as an indication of social fear, although gaze duration and fixation count showed an opposite response to oxytocin treatment in the two studies. Oxytocin is known to attenuate fear responses in many species including humans (Domes et al., 2007) thus the elimination of gaze bias toward the eye region of happy (i.e., less threatening) faces may be based on the anxiety-relieving effects of this neuropeptide. At the same time oxytocin was insufficient to eliminate the attention-getting effects of eye-region of angry faces which still kept some of its fear-evoking potential.

Previous studies have shown that male and female dogs might react differentially (or to a different magnitude) to intranasal oxytocin treatment (Oliva, Kovács). Furthermore the effect of intranasal oxytocin is also modulated by dogs’ breed (and within breeds individuals with different OXTR genotype also react differently; Kovács). The present study did not address such individual variability, but further studies might investigate these together with differences in e.g., subjects’ personality.

In sum our results revealed that compared to humans there are both similarities and differences in how oxytocin influences the way dogs visually explore human emotional faces. The present study also points to limitations of the sequential picture viewing paradigm for assessing cognitive- and attentional processes in dogs and highlights methodological challenges related to eye-tracking data collection.

## Ethics Statement

Ethical approval was obtained from the National Animal Experimentation Ethics Committee (Ref. No. XIV-I-001/531-4-2012). Research was done in accordance with the Hungarian regulations on animal experimentation and the Guidelines for the use of animals in research described by the Association for the Study Animal Behavior (ASAB).

## Author Contributions

AH and JT conceived the experiment. AK, AH, OK and BM performed the experiments. AK and AH analyzed the data and wrote the manuscript. AK and JT secured funding. JT supervised the project.

## Conflict of Interest Statement

The authors declare that the research was conducted in the absence of any commercial or financial relationships that could be construed as a potential conflict of interest.
