# Supplementary material for: The Way Dogs (Canis familiaris) Look at Human Emotional Faces Is Modulated by Oxytocin. An Eye-Tracking Study
Source: Front Behav Neurosci. 2017 Oct 31;11:210. doi: 10.3389/fnbeh.2017.00210 (PMC5671652; doi:10.3389/fnbeh.2017.00210)
Supplement: Supplementary file 1 [file Data_Sheet_1.doc]

**Supplementary material**

Kis, Hernádi et al.

The way dogs *(Canis familiaris)* look at human emotional faces is modulated by oxytocin. An eye-tracking study

**Supplementary table for the main results**

Full statistical models for results reported in the main text are given below. OTPL: oxytocin/placebo pre-treatment; HA: happy/angry emotional expression.

| Source | Numerator df | Denominator df | F | Sig. |
| --- | --- | --- | --- | --- |
| Intercept | 1 | 84 | 67,539 | 0 |
| OTPL | 1 | 84 | 0,008 | 0,929 |
| HA | 1 | 84 | 1,976 | 0,163 |
| order | 1 | 84 | 0,112 | 0,739 |
| OTPL * HA | 1 | 84 | 4,676 | **0,033** |
| OTPL * order | 1 | 84 | 0,723 | 0,398 |
| HA * order | 1 | 84 | 2,218 | 0,14 |
| OTPL * HA * order | 1 | 84 | 1,345 | 0,25 |
| Dependent Variable: rel_face. | | |  |  |

| Source | Numerator df | Denominator df | F | Sig. |
| --- | --- | --- | --- | --- |
| Intercept | 1 | 84 | 34,796 | 0 |
| OTPL | 1 | 84 | 0,386 | 0,536 |
| HA | 1 | 84 | 0,816 | 0,369 |
| order | 1 | 84 | 0,207 | 0,651 |
| OTPL * HA | 1 | 84 | 1,915 | 0,17 |
| OTPL * order | 1 | 84 | 1,836 | 0,179 |
| HA * order | 1 | 84 | 7,372 | 0,008 |
| OTPL * HA * order | 1 | 84 | 1,554 | 0,216 |
| Dependent Variable: rel_eye. | | |  |  |

| Source | Numerator df | Denominator df | F | Sig. |
| --- | --- | --- | --- | --- |
| Intercept | 1 | 84 | 18,333 | 0 |
| OTPL | 1 | 84 | 0,056 | 0,814 |
| HA | 1 | 84 | 1,12 | 0,293 |
| order | 1 | 84 | 3,412 | 0,068 |
| OTPL * HA | 1 | 84 | 0,517 | 0,474 |
| OTPL * order | 1 | 84 | 0,159 | 0,691 |
| HA * order | 1 | 84 | 7,547 | **0,007** |
| OTPL * HA * order | 1 | 84 | 0,087 | 0,769 |
| Dependent Variable: rel_mouth. | | | |  |

| Source | Numerator df | Denominator df | F | Sig. |
| --- | --- | --- | --- | --- |
| Intercept | 1 | 84 | 7,592 | 0,007 |
| OTPL | 1 | 84 | 3,302 | 0,073 |
| HA | 1 | 84 | 0,436 | 0,511 |
| order | 1 | 84 | 0,013 | 0,908 |
| OTPL * HA | 1 | 84 | 1,145 | 0,288 |
| OTPL * order | 1 | 84 | 0,018 | 0,893 |
| HA * order | 1 | 84 | 0,051 | 0,823 |
| OTPL * HA * order | 1 | 84 | 0,257 | 0,613 |
| Dependent Variable: rel_neck. | | | |  |

| Source | Numerator df | Denominator df | F | Sig. |
| --- | --- | --- | --- | --- |
| Intercept | 1 | 84 | 10,737 | 0,002 |
| OTPL | 1 | 84 | 0,381 | 0,539 |
| HA | 1 | 84 | 0,198 | 0,657 |
| order | 1 | 84 | 3,94 | **0,05** |
| OTPL * HA | 1 | 84 | 1,92 | 0,17 |
| OTPL * order | 1 | 84 | 0,034 | 0,853 |
| HA * order | 1 | 84 | 1,893 | 0,173 |
| OTPL * HA * order | 1 | 84 | 0,207 | 0,65 |
| Dependent Variable: rel_forehead. | | | |  |

Details of the rank tests (gaze preference score) are given below:

| **Placebo** | **Happy** |
| --- | --- |
| Ranks |  |
|  | Mean Rank |
| rank_eye | 3,28 |
| rank_mouth | 2,39 |
| rank_forehead | 2,24 |
| rank_neck | 2,09 |
| Test Statistics | |
| N | 23 |
| Chi-Square | 19,705 |
| df | 3 |
| Asymp. Sig. | 0 |
| Friedman Test | |

| **Placebo** | **Angry** |
| --- | --- |
| Ranks |  |
|  | Mean Rank |
| rank_eye | 3,28 |
| rank_mouth | 2,5 |
| rank_forehead | 2,2 |
| rank_neck | 2,02 |
| Test Statistics | |
| N | 23 |
| Chi-Square | 19,123 |
| df | 3 |
| Asymp. Sig. | 0 |
| Friedman Test | |

| **Oxytocin** | **Happy** |
| --- | --- |
| Ranks |  |
|  | Mean Rank |
| rank_eye | 2,96 |
| rank_mouth | 2,52 |
| rank_forehead | 2,26 |
| rank_neck | 2,26 |
| Test Statistics | |
| N | 23 |
| Chi-Square | 6,706 |
| df | 3 |
| Asymp. Sig. | 0,082 |
| Friedman Test | |

| **Oxytocin** | **Angry** |
| --- | --- |
| Ranks |  |
|  | Mean Rank |
| rank_eye | 2,98 |
| rank_mouth | 2,54 |
| rank_forehead | 2,28 |
| rank_neck | 2,2 |
| Test Statistics | |
| N | 23 |
| Chi-Square | 9,333 |
| df | 3 |
| Asymp. Sig. | 0,025 |
| Friedman Test | |

| **Happy** |  |  |  |  |
| --- | --- | --- | --- | --- |
| Test Statistics | |  |  |  |
|  | rank_eye | rank_mouth | rank_forehead | rank_neck |
| Mann-Whitney U | 207,5 | 251 | 263 | 247 |
| Wilcoxon W | 483,5 | 527 | 539 | 523 |
| Z | -1,327 | -0,304 | -0,034 | -0,398 |
| Asymp. Sig. (2-tailed) | 0,184 | 0,761 | 0,973 | 0,691 |
| Grouping Variable: OTPL | | |  |  |

| **Angry** |  |  |  |  |
| --- | --- | --- | --- | --- |
| Test Statistics | |  |  |  |
|  | rank_eye | rank_mouth | rank_forehead | rank_neck |
| Mann-Whitney U | 210 | 264 | 200,5 | 228,5 |
| Wilcoxon W | 486 | 540 | 476,5 | 504,5 |
| Z | -1,263 | -0,011 | -1,464 | -0,825 |
| Asymp. Sig. (2-tailed) | 0,206 | 0,991 | 0,143 | 0,409 |
| Grouping Variable: OTPL | | |  |  |
